# Heterobivalent Dual-Target Peptide for Integrin-α_v_β_3_ and Neuropeptide Y Receptors on Breast Tumor

**DOI:** 10.3390/ph17101328

**Published:** 2024-10-04

**Authors:** Aryel H. Ferreira, Caroline C. Real, Osvaldo Malafaia

**Affiliations:** 1 MackGraphe-Mackenzie Institute for Research in Graphene and Nanotechnologies, Mackenzie Presbyterian University, São Paulo 01302-907, Brazil; 2 Mackenzie Evangelical College of Paraná, Mackenzie Presbyterian University, Curitiba 80730-000, Brazil; 3 Nuclear and Energy Research Institute, Instituto de Pesquisas Energéticas e Nucleares da Comissão Nacional de Energia Nuclear—São Paulo (IPEN-CNEN/SP), São Paulo 05508-000, Brazil; 4 Department of Nuclear Medicine and PET Center, Aarhus University Hospital, 8200 Aarhus, Denmark

**Keywords:** radiolabeled peptide, heterobivalent peptide, neuropeptide Y, RGD peptide, molecular imaging, radiopharmaceuticals, technetium-99m

## Abstract

**Background/Objectives:** Heterodimer peptides targeting more than one receptor can be advantageous, as tumors can simultaneously express more than one receptor type. For human breast cancer, a promising biological target is tumor angiogenesis through α_v_β_3_ integrin expression. Another promising target is Neuropeptide Y receptors, considering Y_1_R is overexpressed in 90% of human breast tumors. This article details the development and preclinical evaluation, both in vitro and in vivo, of a novel heterodimer peptide dual-receptor-targeting probe, [^99m^Tc]HYNIC-cRGDfk-NPY, designed for imaging breast tumors. **Methods:** Female BALB/c healthy mice were used to perform biodistrubution studies and female SCID mice were subcutaneously injected with MCF-7 and MDA-MB-231 tumor cells. [^99m^Tc]HYNIC-cRGDfk-NPY was intravenously administered to the mice, followed by ex vivo biodistribution studies and small-animal SPECT/CT imaging. Nonspecific tracer uptake in both models was determined by coinjecting an excess of unlabeled HYNIC-cRGDfk-NPY (100 µg) along with the radiolabeled tracer. **Results:** Imaging and biodistribution data demonstrate good uptake to estrogen receptor-positive (MCF-7) and triple-negative (MDA-MB-231) tumor models. The in vivo tumor uptakes of radiolabeled conjugate were 9.30 ± 3.25% and 4.93 ± 1.01% for MCF-7 and MDA-MB231, respectively. The tumor/muscle ratios were 5.65 ± 0.94 for the MCF-7 model and 7.78 ± 3.20 for MDA-MB231. **Conclusions:** [^99m^Tc]HYNIC-cRGDfk-NPY demonstrated rapid blood clearance, renal excretion, and in vivo tumor uptake, highlighting its potential as a tumor imaging agent.

## 1. Introduction

Radiolabeled peptides have been used in Nuclear Medicine clinical practice to target specific receptors overexpressed in tumors, both for diagnosis or treatment of tumors or metastasis, as, depending on the radionuclide used, the same chemical structure can be used for both applications [1,2,3]. However, these radiolabeled monovalent peptides can only mark one type of target receptor and can only visualize tumors that express a specific receptor [4,5].

Heterodimer peptides targeting more than one receptor target may be advantageous as tumors can simultaneously express more than one receptor type [6]. The design of heterodimer peptides can significantly increase the avidity and specificity of the contrast agent due to simultaneous binding to more than one type of receptor or at least one receptor independently, compared to their corresponding monospecific counterparts [5].

Furthermore, statistically, the uptake probability of a heterodimer peptide increases considering the total number of receptors expressed on the cell surface [5]. Heterodimer peptides also present advantages in cases of heterogeneous expression of receptors in tumor lesions, that vary among individuals [7]. In cases where there are differences in receptor density, some tumor lesions may go unnoticed on the images due to the non-homogeneous distribution of receptors. This situation can be circumvented with heterodimer peptides [8].

In this context, for human breast cancer, a promising biological target is tumor angiogenesis, where newly formed blood vessels show high expression of α_v_-integrins, which are less expressed in mature vasculature [9]. Peptides containing the sequence arginine–glycine–aspartic acid (RGD) are known to target α_v_β_3_ receptors, that are strongly overexpressed on the activated endothelium of new blood vessels [10]. Recently, a heterobivalent peptide containing the RGD sequence was successfully translated into patients with prostate cancer using positron emission tomography combined with computed tomography images (PET/CT), showing much higher tumor visualization sensitivity than the respective monovalent bombesin peptide to target Gastrin-Releasing Peptide Receptor [11].

Another promising target in breast cancer is Neuropeptide Y receptors (Y_1_R and Y_2_R), considering that overexpression was found in 85% of breast cancer patients, with Y_1_R expressed in most breast cancers and Y_2_R in only 24%. Moreover, Y_1_R is overexpressed in 90% of human breast tumors and 100% of detected metastatic tumors. The neuropeptide Y (NPY) is an essential neurotransmitter in the central and peripheral nervous system and is involved in the development of specific tumors [12,13,14]. Along with other hormone peptide receptors overexpressed in human cancer, NPY favors in vivo tumor labeling for diagnostic and therapeutic purposes.

A heterodimer molecule consisting of cyclic RGD and NPY analog motifs in a single probe is an attractive approach, as it targets two receptors that, although expressed differently, play important roles in breast cancer. The cell lines selected for this study exhibit distinct expression profiles of NPY and α_V_β_3_ receptors, making them ideal models to evaluate the synergistic effect of peptide heterodimerization. Specifically, the human breast cancer cell line MCF-7 expresses both NPY receptors and α_V_β_3_, while MDA-MB-231 cells are predominantly α_V_β_3_-positive but show low expression of NPY receptors [4,15]. These differences provide a valuable opportunity to explore how dual receptor binding could enhance the probe’s targeting efficiency. The promising pharmacological properties of both peptides prompted us to develop a radiolabeled heterodimer to enhance tumor uptake and bioavailability and capitalize on potential efficacy to successfully deliver contrast agents and diagnostic radionuclides. The current paper describes the procedure for radiolabeling cRDGfK-NPY with technetium-99m (^99m^Tc), the in vitro parameters, and, as a proof of concept, the in vivo imaging biodistribution investigations.

## 2. Results

### 2.1. Radiolabeling of HYNIC-cRDGfK-NPY Peptide

**Figure 1:**
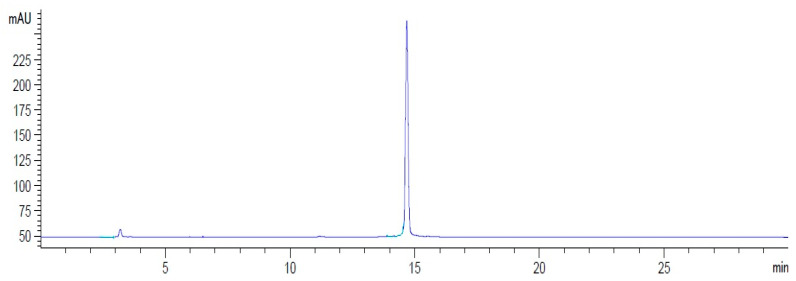
Reverse-phase HPLC profile of [^99m^Tc]HYNIC-cRDGfk-NPY.

Radiolabeling of the HYNIC-cRDGfK-NPY conjugate was performed with a specific activity of 161.22 MBq/µM. The radiochemical purity was evaluated by ITLC using a solvent system of MEK and 50% ACN solution. In MEK, ^99m^TcO_4_^−^ had a retention factor (R_f_) of 1, whereas for ^99m^TcO_2_ and the radiolabeled conjugate, the R_f_ was 0. In the ACN solution, ^99m^TcO_4_^−^ and the radiolabeled conjugate displayed an Rf of 1 and ^99m^TcO_2_ an R_f_ of 0. The radiochemical purity was 97.4 ± 1.4%, and the free pertechnetate (TcO_4_^−^) level was approximately 1.4% ± 0.8%, whereas colloidal technetium was 1.2 ± 0.7%. The ITLC findings were confirmed by HPLC (Figure 1) with a single peak for [^99m^Tc]HYNIC-cRDGfk-NPY and a retention time of 14.9 min. Pertechnetate had a retention time of 3.9 min under these HPLC conditions. The lipophilicity of [^99m^Tc]HYNIC-cRDGfk-NPY was determined based on the corresponding distribution between n-octanol and water. The mean value of the partition coefficient logarithm (Log *p*) was −2.58 ± 0.30, which is within the hydrophilic range (negative values).

### 2.2. [^99m^Tc]HYNIC-cRDGfk-NPY Biodistribution in Balb/c Healthy Mice

**Figure 2:**
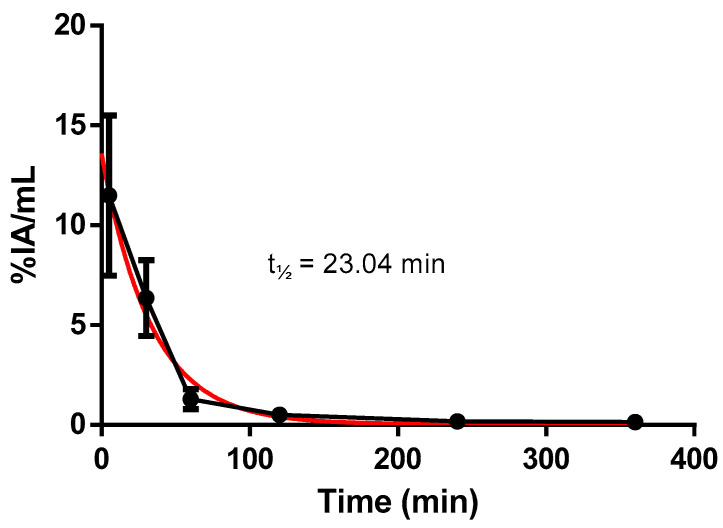
Blood clearance in Balb/c healthy mice. The values are expressed in %IA/mL (0.05 mL/18.5 MBq). Blood half-life = 23.04 min. (Red line—Exponential decay).

**Figure 3:**
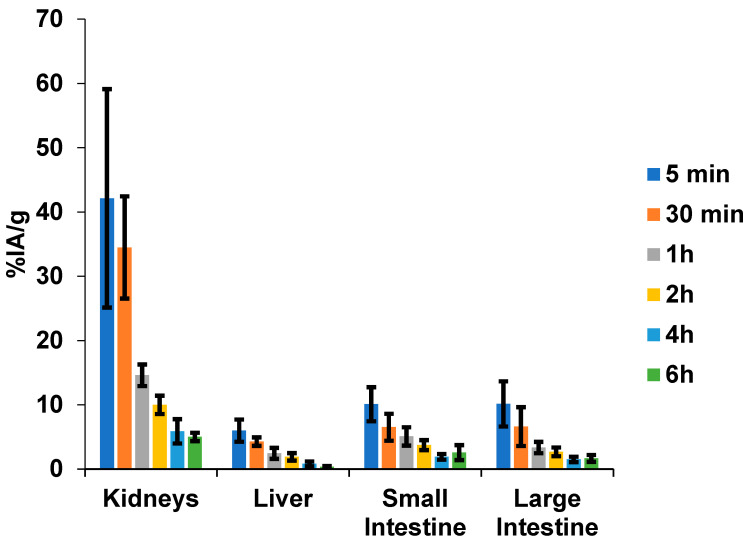
Excretion organ uptake of [^99m^Tc]HYNIC-cRDGfk-NPY in Balb/c healthy mice (0.05 mL/18.5 MBq). The radioactivity in the intestines was evaluated after thoroughly removing the luminal contents.

The ex vivo biodistribution analysis performed with healthy Balb/c mice is presented in Figure 2 and Figure 3, and Table 1. Values are expressed as a percentage of injected activity per gram (%IA/g) of tissue and per milliliter (%IA/mL) of blood. The biodistribution data indicate rapid blood clearance, with a decrease in activity of approximately 90% within 1 h (Figure 2).

The urinary system is the primary excretory pathway, with predominant renal uptake. However, hepatobiliary excretion was also detected, as indicated by the amount remaining in the intestinal tract (Figure 3).

Among other organs, the highest uptakes of the radiotracer were observed in the lungs, followed by the stomach and spleen, at 5 and 30 min p.i. (Table 1). The uptake of the radiotracer diminished to less than 3% one hour post-injection, except in the stomach and spleen, where it remained at around 2% for longer durations. Moderate uptake was found in the heart and bone at the first three study time points, and low uptake was observed in the brain, indicating that the radiotracer did not cross the blood–brain barrier.

### 2.3. In Vitro Cell Binding

**Figure 4:**
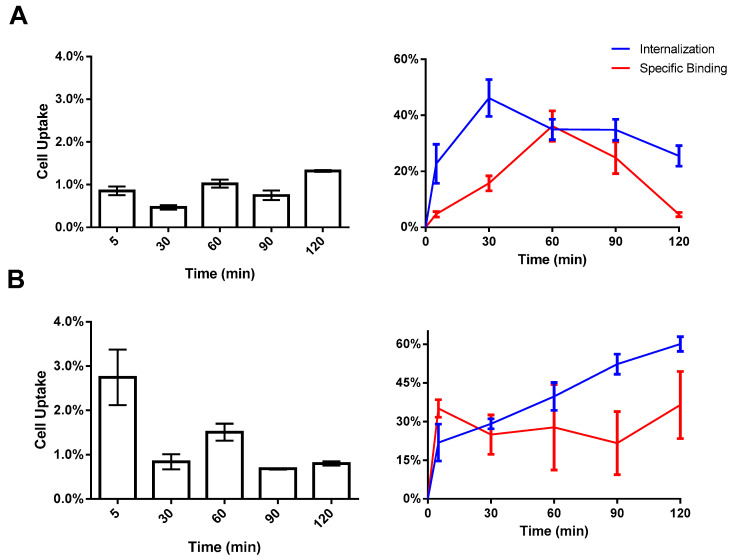
In vitro cell uptake: binding and internalization of [^99m^Tc]HYNIC-cRGDfk-NPY in (**A**) MDA-MB231 cells and (**B**) MCF-7 cells at different time intervals.

The tumor-targeting properties of [^99m^Tc]HYNIC-cRGDfk-NPY were examined in vitro using MDA-MB-231 and MCF-7 breast cancer cell lines. The cellular uptake and internalization behavior of the radiotracer over time is presented in Figure 4. Following 5 min of incubation with [^99m^Tc]HYNIC-cRGDfk-NPY, around 0.86 ± 0.10% of the total added activity was bound to MDA-MB231 cells, increasing to 1.32 ± 0.02% after 2 h incubation. However, after 1 h of incubation, better performance-related internalization and specific binding were observed; approximately 34.95 ± 3.62% was internalized, and 36.16 ± 3.62% was bound to the cell membrane, representing approximately 70% of the total cell-associated activity. For MCF-7 cells, approximately 2.75 ± 0.63% of the total activity of [^99m^Tc]HYNIC-cRGDfk-NPY was bound after 5 min. The best value was reached at 1.5 h, when around 74% of total cell-associated activity was observed, considering internalization and specific binding ratios.

### 2.4. Biodistribution in Nude Mice Bearing MCF-7 and MDA-MB231 Human Breast Cancer Xenografts

**Figure 5:**
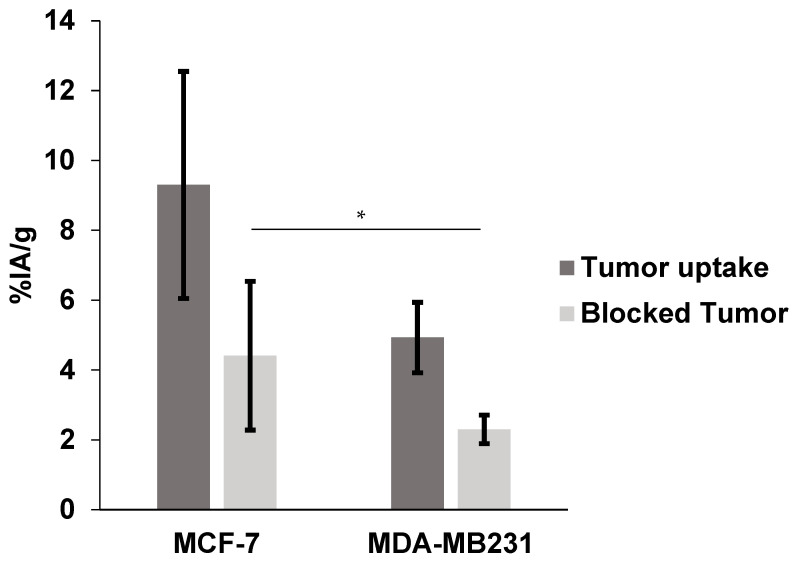
[^99m^Tc]HYNIC-cRGDfk-NPY in vivo uptake. Tumor and blocked tumor of SCID mice bearing MCF-7 and MDA-MB231 at 1 h after injection (* *p* < 0.05).

**Figure 6:**
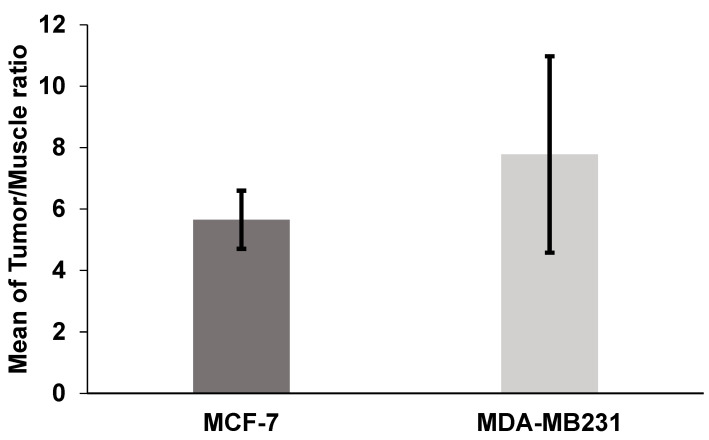
Mean tumor-muscle uptake ratio in MCF-7 and MDA-MB231 tumor-bearing SCID mice (n = 5) at 1 h after intravenous (IV) injection of [^99m^Tc]HYNIC-cRGDfk-NPY.

**Figure 7:**
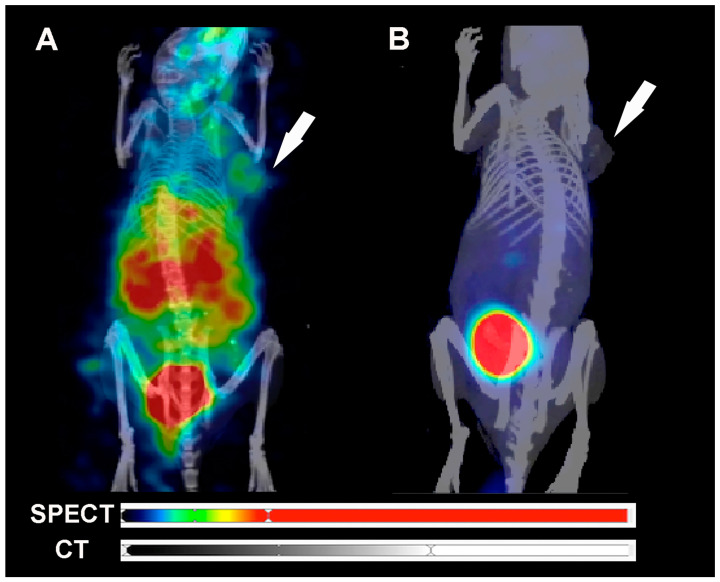
Maximum intensity projection (MIP) SPECT/CT images of [^99m^Tc]HYNIC-cRGDfk-NPY. (**A**) Tumor and (**B**) blocked tumor of SCID mice bearing MDA-MB231 model 1 h post-injection.

To evaluate the uptake of the radiolabeled peptide by breast tumors, additional biodistribution studies were performed in SCID mice bearing MCF-7 and MDA-MB-231 xenografts (Table 2). Results compared to healthy mice at 1 h p.i. showed similar patterns of low liver and high kidney uptakes and rapid clearance from the blood. The tumor uptakes of the radiolabeled conjugate were 9.30 ± 3.25% for MCF-7 and 4.93 ± 1.01% for MDA-MB-231 (Figure 5). The tumor-to-muscle ratios were 5.65 ± 0.94 for the MCF-7 model and 7.78 ± 3.20 for the MDA-MB-231 model (Figure 6). Additionally, in the blocking study, tumor uptake was reduced from 9.30 ± 3.25% to 4.41 ± 2.13% for MCF-7 (*p* > 0.05) and from 4.93 ± 1.01% to 2.30 ± 0.41% for MDA-MB-231 (*p* < 0.05) when a 500-fold molar excess of the cold peptide was injected 30 min before the radioconjugated peptide (Figure 7).

### 2.5. SPECT/CT Image and Scintigraphy Image

**Figure 8:**
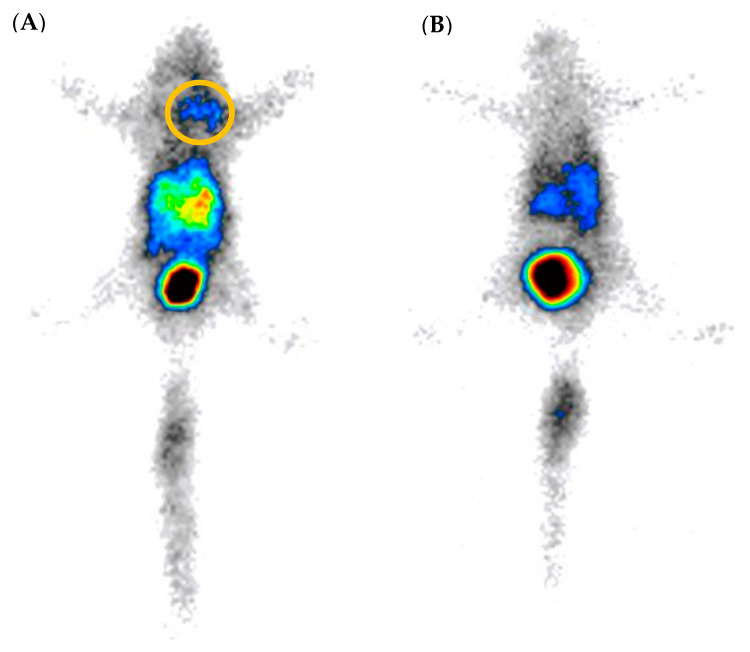
Scintigraphy image of [^99m^Tc]-HYNIC-cRDGfk-NPY. (**A**) Tumor and (**B**) blocked tumor of SCID mice bearing MCF-7 human breast carcinoma 1 h after injection.

In vivo biodistribution, SPECT/CT, and scintigraphy images of [^99m^Tc]HYNIC-cRGDfk-NPY in MDA-MB-231 and MCF-7 tumor-bearing SCID mice 1 h p.i. are shown in Figure 7 and Figure 8. The images show that the probe is detected in the kidneys and bladder due to renal elimination, confirming the ex vivo biodistribution results. Additionally, the tumor was visualized by SPECT and scintigraphy imaging at 1 h p.i. (Figure 7 and Figure 8 and Supplementary Material—Video S1: Blocked tumor; Video S2: Tumor uptake).

## 3. Discussion

Nuclear medicine offers simple and non-invasive methods for early cancer diagnosis and treatment, through the utilization of molecular targeting probes [16,17]. The α_V_β_3_ integrin is well-known to have a crucial role in tumor progression and angiogenesis of a variety of human cancers [18]. The α_V_β_3_ is overexpressed in breast cancer and activates endothelial cells during pathological angiogenesis, giving them the ability to modulate cell adhesion, migration, and invasion through new blood vessels, enhancing the metastatic process [19,20]. Apart from the NPY receptor, which is overexpressed in 90% of human breast tumors, α_V_β_3_ became an important target for developing new imaging probes. The RGD peptide is a small peptide that is able to bind α_V_β_3_ integrin and the NPY analog sequence could bind to the NPY receptors.

The final goal of the current study was to develop a novel, non-invasive imaging agent based on heterobivalent peptide labeled with ^99m^Tc to introduce a potential for in vivo targeting of tumors with overexpression of α_V_β_3_ and/or NPY receptors. Several chelating agents, such as Diethylenetriaminepentaacetic acid (DTPA), mercaptoacetyltriglycine (MAG3), and precursors, such as Tricarbonyl Complex [^99m^Tc(CO)_3_]^+1^ can coordinate the radionuclide technetium-99m (^99m^Tc) for applications in cancer diagnosis by nuclear medicine techniques [21,22]. In the current study, the strategy to use HYNIC as a chelator was chosen due to simple, high-yield labeling and well-known stability in a biological system since some blood proteins possess metal-complexing properties, which lead to transchelation of ^99m^Tc atoms to these proteins [23]. Tricine and EDDA were selected as co-ligands because they provide acceptable radiolabeling efficacy. This approach generated a radiolabeled peptide with high radiochemical purity (>97%).

The in vivo study in normal mice showed the probe interaction with blood, normal tissues, and organs. [^99m^Tc]-HYNIC-cRDGfk-NPY presented rapid blood clearance and was excreted primarily through the renal system, with secondary clearance occurring via the hepatobiliary system, which agrees with the hydrophilic nature of radiolabeled compounds found in the partition coefficient studies. The increased stomach uptake could be associated with low radiochemical purity in studies involving radiolabeling with ^99m^Tc [24,25]. However, the gastrointestinal tract has a high expression level of NPY receptors [26]. Previous studies report that NPY peptide is related to stomach physiological functions, such as appetite regulation and obesity induction [27,28]. High uptake in the stomach and intestines was noted, suggesting that NPY inhibits contractile activity and induces slow motility and decreased intestinal fluid secretion [29]. Data analysis also reveals important retention in the spleen at early times. This result also reflects the high number of cells carrying NPY receptors in this tissue. Regions with high NPY receptor density were found in the splenic hilum of rats, dogs, and pigs, in the nervous plexuses surrounding the central arteries of the white pulp of this organ, as well as in splenic lymphocytes [30,31,32].

It has previously been reported that different subtypes of NPY receptors are expressed in several human cancers, including breast carcinomas, ovarian sex cord–stromal tumors, adrenal cortical tumors, glioblastomas, nephroblastomas, renal cell carcinomas, Ewing sarcoma tumors, gastrointestinal stromal tumors, and testicular tumors [33]. In addition, integrin α_v_β_3_ is overexpressed in the neovasculature from breast cancer [34].

The current study compared the tendency of [^99m^Tc]-HYNIC-cRDGfk-NPY complex in binding to MCF-7 and MDA-MB231 cell lines. These cell lines have different levels for both NPY receptor and α_V_β_3_ expression; therefore, they are ideal models for determining the synergistic effect of peptide heterodimerization. Undoubtedly, it would also have been possible to use different models that express only α_V_β_3_ or NPY receptors to demonstrate respective target receptors. Furthermore, a cell line that concomitantly expresses both receptors is far more advantageous in showing the potential beneficial effects of heterodimerization. The human breast cancer cell line MCF-7 was described to express both NPY(Y_1_)R and α_V_β_3_, whereas MDA-MB-231 cells were described to be α_V_β_3_ positive but expressing the NPY receptors to a very low extent [4,15]. This means that the heterobivalent ligand design could maximize the radiotracer uptake by targeting multiple receptors, even at different levels of expression between estrogen-positive and triple-negative breast cancer cells. Furthermore, it was described that breast cancer cells could express both receptors to a much higher extent in vivo than under in vitro conditions; this might explain the differences in uptake in in vitro and in vivo experiments [35].

The higher in vivo tumor uptake of [^99m^Tc]-HYNIC-cRDGfk-NPY resulted in higher overall tumor-to-muscle ratios, approximately 5.65 ± 0.94 and 7.78 ± 3.20, for MCF-7 and MDA-MB231, respectively, which should translate into images with higher contrast. The uptake in non-target tissues was similar in the healthy and mice models. The binding specificity of tested heterobivalent peptides was demonstrated both in vitro and in vivo by blocking receptors with an excess of non-labeled peptides. The radiolabeled signal was considerably suppressed in tumors and other tissues where the presence of NPY receptor and αvβ3 integrin is expected. Co-injection with cold HYNIC-cRDGfk-NPY reduced the accumulation in the MCF-7 model from 9.30 ± 3.25 to 4.41 ± 2.13%IA/g (change ns, *p* > 0.05) and in the MDA-MB231 model from 4.93 ± 1.01 to 2.30 ± 0.41%IA/g (significant change, *p* < 0.05).

## 4. Materials and Methods

**Figure 9:**
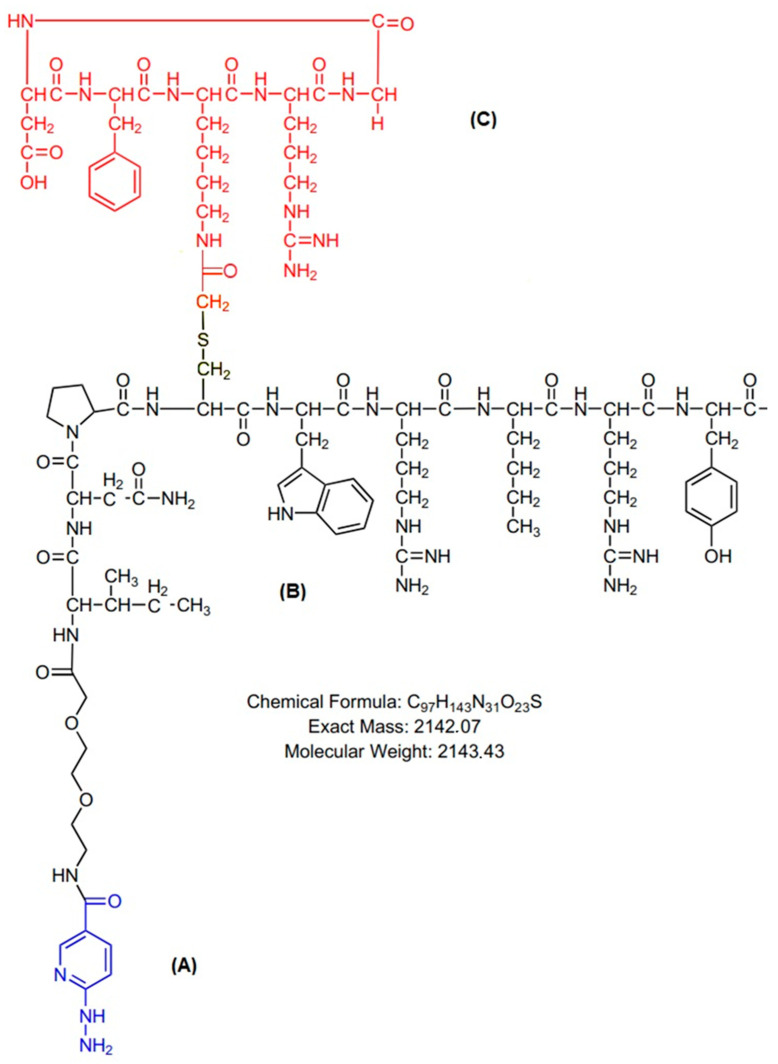
Structural formula of HYNIC-cRDGfK-NPY molecular weight: 2143.43, (**A**) HYNIC; (**B**) NPY analog; (**C**) RGD cyclic peptide. Sequence: HYNIC-O_2_Oc-Ile-Asn-Pro-Cys[cyclo(-Arg-Gly-Asp-dPhe-Lys(COCH2)-)]-Trp-Arg-Nle-Arg-Tyr-NH_2_.

Technetium-99m was eluted in 6 milliliters of isotonic saline solution from an alumina-based ^99^Mo/^99m^Tc generator, supplied locally by the Radiopharmacy Center of the Nuclear and Energy Research Institute (IPEN/CNEN)—Sao Paulo, Brazil. All other reagents were purchased from Sigma-Aldrich Brazil Ltda and used without further purification. The heterodimer peptide-conjugated cyclic arginine–glycine–aspartic acid and NPY analog [abbreviated (HYNIC-cRDGfK-NPY] (MW 2143.43, peptide purity > 90%) was designed by the author, synthesized, and purchased from piChem (Raaba-Grambach, Austria) as a lyophilized powder (Figure 9).

### 4.1. ^99m^Tc Radiolabeling of HYNIC-cRDGfK-NPY

Radiolabeling processes were performed using the method described by Descristoforo et al. [36]. The conjugated peptide HYNIC-cRDGfK-NPY, in salt form, was dissolved in water (1 mg/mL). Radiolabeling was performed by adding 0.5 mL of Na^99m^TcO_4_ (740 MBq) to a sealed reaction vial containing 20 mg of tricine and 5 mg of EDDA (ethylenediamine-N,N′-diacetic acid) dissolved in 500 μL of 0.1 M of nitrogenated phosphate buffer solution, 10 μL of conjugated peptide solution (μL/mL), and 5 μL (22 mM) of SnCl_2_·2H_2_O solution in 0.1 N HCl (nitrogen-purged). The reaction was induced by heating the mixture to 100 °C for 20 min.

### 4.2. Radiochemical Control

The radiochemical purity was determined by instant thin-layer chromatography on silica gel strips (ITLC-SG) using different mobile phases, (1) methyl ethyl ketone (MEK) and (2) acetonitrile (CH_3_CN)/water (50/50) [37]. The radiolabeled conjugate was also characterized by reverse-phase, high-performance liquid chromatography analysis on an Infinity Better/Agilent Technologies HPLC System. The HPLC solvents consisted of H_2_O, which contained 0.1% trifluoroacetic acid (solvent A), and CH_3_CN, which contained 0.1% trifluoroacetic acid (solvent B). The HPLC gradient system began with a solvent composed of 95% A and 5% B, which was followed by a linear gradient of 30% A and 70% B from 0 to 25 min, and 5% A and 95% B from 25 to 30 min [37].

### 4.3. Partition Coefficient (P) Determination

The lipophilicity of the complex was determined using solvent extraction in octanol/water (1:1). A fraction (100 µL) of ^99m^Tc-HYNIC-cRDGfK-NPY was added to this mixture, which was then vigorously vortexed for one minute at room temperature and centrifuged at 1500 rpm for 15 min. Equal samples were collected from both aqueous/organic phases, and their radioactivity was measured in a gamma counter (2470 Automatic Gamma Counter—PerkinElmer, Waltham, MA, USA) to determine the log *p* values (n = 5) [38].

### 4.4. Cell Culture

MCF-7 (ER-positive) and MDA-MB-231 (triple-negative) human breast cancer cell lines were incubated at 37 °C in a humidified atmosphere containing 5% CO_2_ and maintained in culture using RPMI 1640 supplemented with 10% fetal bovine serum (Sigma-Aldrich; Darmstadt, Germany) and 1% penicillin/streptomycin. Cells were grown to 75% confluence and then harvested with trypsinization (0.04% trypsin/EDTA). After centrifugation (151× *g* for 5 min), the cells were resuspended either in a fresh medium containing 0.1% bovine serum albumin (BSA) for in vitro receptor binding assay or pH 7.4 phosphate-buffered saline (PBS) for tumor inoculation in animals.

### 4.5. In Vitro Specific Binding to MCF7 and MDA-MB-231 Cells

The specific binding of the radiopeptide to MCF-7 and MDA-MB-231 cells was evaluated in 6-well adherent plates containing 10^6^ cells per well. For the specific binding study (unblock), a supplemented RPMI 16 medium containing 100 µL of the ^99m^Tc-HYNIC-cRDGfK-NPY was added to the wells. On the other hand, for the non-specific binding study (block), an excess of the respective non-radiolabeled peptide (1 μM, competitor) was added to the supplemented RPMI 16 medium containing the radiopeptide in each well. In both cases, the plates were incubated at 37 °C in a humidified atmosphere containing 5% CO_2_, for various periods (5 to 120 min). The supernatant was collected, and the cell surface-bound radioligand was removed by acid wash buffer (50 mM glycine buffer pH 2.8, 0.1 M NaCl) at room temperature for 5 min. The internalized radioligand was determined by solubilization of the cells with 1 N NaOH [38]. Results were expressed as a percentage of total radioactivity, considering cell surface-bound and internalized activity.

### 4.6. Breast Cancer Tumor-Bearing Animal Model

The animals used in this experiment were provided by the Nuclear and Energy Research Institute (IPEN/CNEN-SP) vivarium. Every effort was made to minimize animal suffering per International Ethical Guidelines. The experimental protocols used in this study were approved by the Scientific Ethics Committee of the IPEN/CNEN-SP. Adult female SCID mice were maintained under controlled environmental conditions (12 h light-dark cycle; constant temperature 21 ± 2 °C), with free access to water and food. Suspensions of cells (5 × 10^6^ in 0.1 mL) were subcutaneously injected into the upper back regions of the mice.

### 4.7. Ex Vivo Biodistribution Studies

The ex vivo biodistribution of the radiolabeled peptide (50 μL/37 MBq) was evaluated at 5 min, 30 min, 1 h, 2 h, 4 h, and 6 h after IV injection in control and tumor-bearing animals (n = 5 per group, per time point). Animals were deeply anesthetized and euthanized at the corresponding biodistribution time point. Blood, brain, spleen, heart, stomach, liver, lungs, kidneys, pancreas, muscle, bone, small intestine, and large intestine were collected and weighed. The radioactivity of each organ/tissue was then determined using the above-mentioned automatic gamma counter. The radioactivity was evaluated as the percentage of injected activity per gram of organ/tissue (%IA/g), using the same injected dose activity as a standard. Receptor-blocking studies in tumor models were also carried out by co-administration of 100 μg of cold-conjugated peptide along with radiopeptide.

### 4.8. SPECT/CT Image and Scintigraphy Image

SPECT/CT images were acquired for the MDA-MB231 model in a dedicated small animal scanner (MicroPET/SPECT/CT Albira-Bruker). All procedures were performed with the animal under anesthesia (2–3% isoflurane in 100% oxygen). ^99m^Tc-HYNIC-cRDGfK-NPY, 37 MBq in 0.1 mL, was administrated intravenously into the tail vein. After 1 h post-injection, the animal was positioned with the tumor region in the center of the SPECT scanner field of view. Heating pads were used to maintain the animals’ body temperature, and breath rate was monitored. Before SPECT acquisition, computed tomography (CT) images, 45 kVp, and 360 µA were obtained for 1 min (256 projections, 1.3× magnification). SPECT/CT fused images were analyzed using PMOD^®^ software version 4.0. For the MCF-7 model, anesthetized mice were horizontally placed under a Mediso Imaging System (Budapest, Hungary), employing a low-energy high-resolution collimator. Images were acquired at 1 h p.i., using a 256 × 256 × 16 matrix size, with a 20% energy window set at 140 keV, for 180 s.

### 4.9. Statistical Analysis

The Student’s *t*-test was used to compare the means of the two groups. A significance interval of 95% was considered (*p*-value < 0.05). The data were analyzed using GraphPad Prism v.8.0.2 software (GraphPad Software Inc., La Joya, CA, USA).

## 5. Conclusions

In this proof-of-concept study, the radiolabeled heterobivalent peptide [^99m^Tc]-HYNIC-cRDGfk-NPY addressed to both NPY and α_v_β_3_ receptors was prepared with high radiochemical purity. The biodistribution of the radioconjugated peptide in healthy mice, and in the MCF-7 and MDA-MB231 xenograft mice models, reveal the renal system as the main route for excretion, followed by the hepatobiliary pathway. The tumor-to-muscle ratio of this new compound exhibits specificity and affinity to target MCF-7 and MDA-MB 231 tumors. In summary, this study suggests the potential of heterobivalent radioligand [^99m^Tc]-HYNIC-cRDGfk-NPY to target breast tumors by targeting more than one biomarker as an agent for SPECT imaging.

## Figures and Tables

**Table 1:** Ex vivo biodistribution data obtained after intravenous injection of [^99m^Tc]HYNIC-cRGDfk-NPY in Balb/c healthy mice (%IA/g ± SD n = 5 per time).

Organ/Time	5 min	30 min	1 h	2 h	4 h	6 h
Heart	4.30 ± 1.32	3.21 ± 1.22	1.04 ± 0.23	0.55 ± 0.07	0.35 ± 0.12	0.33 ± 0.09
Lung	9.80 ± 3.16	4.80 ± 2.67	2.64 ± 0.83	1.17 ± 0.20	0.63 ± 0.17	1.65 ± 2.02
Spleen	6.06 ± 2.90	6.14 ± 2.29	3.41 ± 1.17	2.22 ± 0.37	1.69 ± 0.34	1.98 ± 0.63
Stomach	8.05 ± 2.56	7.17 ± 1.38	3.97 ± 0.78	2.62 ± 0.68	1.81 ± 0.50	1.79 ± 0.58
Pancreas	3.39 ± 0.92	2.52 ± 0.89	1.17 ± 0.45	0.53 ± 0.10	0.35 ± 0.12	0.34 ± 0.11
Muscle	2.16 ± 0.62	1.50 ± 0.41	0.68 ± 0.33	0.39 ± 0.09	0.22 ± 0.07	0.21 ± 0.03
Bone	3.19 ± 0.76	3.71 ± 1.37	2.52 ± 1.67	0.71 ± 0.22	0.46 ± 0.14	0.54 ± 0.28
Brain	0.37 ± 0.11	0.22 ± 0.15	0.12 ± 0.03	0.07 ± 0.01	0.04 ± 0.01	0.04 ± 0.01

**Table 2:** Biodistribution of [^99m^Tc]HYNIC-cRGDfk-NPY in SCID mice bearing tumor cell lines at 60 min p.i. n = 5 and n = 3 for block (0.1 mL/18.5 MBq) (%IA/g; * %IA/mL).

Organ/Tumor Model	MCF-7		MDA-MB231	
	Non-Block	Block	Non-Block	Block
Blood *	1.66 ± 0.89	0.92 ± 0.07	1.80 ± 1.03	1.40 ± 0.21
Heart	1.53 ± 0.45	0.65 ± 0.24	1.48 ± 0.29	0.74 ± 0.20
Lungs	3.58 ± 1.22	1.59 ± 0.34	3.27 ± 0.47	1.95 ± 0.60
Kidneys	24.55 ± 8.60	20.80 ± 7.02	19.30 ± 2.89	12.99 ± 2.68
Spleen	5.33 ± 1.88	2.53 ± 0.74	5.02 ± 0.63	1.51 ± 0.22
Stomach	6.04 ± 1.15	2.98 ± 0.57	5.63 ± 0.96	2.05 ± 0.33
Pancreas	1.62 ± 0.30	1.12 ± 0.42	1.35 ± 0.35	0.70 ± 0.21
Liver	3.61 ± 0.73	1.84 ± 0.13	3.75 ± 0.51	1.41 ± 0.62
Large Intestine	6.41 ± 1.63	3.91 ± 2.11	5.18 ± 0.87	2.22 ± 0.98
Small Intestine	10.32 ± 2.18	3.77 ± 0.95	8.30 ± 1.36	2.16 ± 0.74
Muscle	1.56 ± 1.41	0.59 ± 0.37	0.88 ± 0.13	0.33 ± 0.02
Bone	1.40 ± 0.72	1.08 ± 0.77	1.22 ± 0.28	0.53 ± 0.16
Brain	0.16 ± 0.06	0.08 ± 0.01	0.18 ± 0.04	0.09 ± 0.01
Tumor	9.30 ± 3.25	4.41 ± 2.13	4.93 ± 1.01	2.30 ± 0.41

## Data Availability

Data are contained within the article.

## Supplementary Materials

The following supporting information can be downloaded at: https://www.mdpi.com/article/10.3390/ph17101328/s1, Video S1: Maximum intensity projection (MIP) SPECT/CT image fusion with 72 angles of [99mTc]HYNIC-cRGDfk-NPY in SCID mice bearing a blocked MDA-MB231 tumor was acquired 1 h after injection; Video S2: Maximum intensity projection (MIP) SPECT/CT image fusion with 72 angles of [99mTc]HYNIC-cRGDfk-NPY in SCID mice bearing MDA-MB231 tumors, acquired 1 h post-injection.
